# Study on the Transport Law and Corrosion Behavior of Sulfate Ions of a Solution Soaking FA-PMPC Paste

**DOI:** 10.3390/ma19010202

**Published:** 2026-01-05

**Authors:** Yuying Hou, Qiang Xu, Tao Li, Sha Sa, Yante Mao, Caiqiang Xiong, Xiamin Hu, Kan Xu, Jianming Yang

**Affiliations:** 1 School of Civil Engineering, SanJiang University, Nanjing 210012, China; hou_yuying@sju.edu.cn (Y.H.); verbundbau@163.com (X.H.); 2 China Construction Third Engineering Bureau Co., Ltd., Wuhan 430205, China; 3 College of Civil Engineering, Yancheng Institute of Technology, Yancheng 224051, China

**Keywords:** potassium magnesium phosphate cement (PMPC), fly ash (FA), transport law of sulfate ions, strength, deformation

## Abstract

To study the sulfate corrosion behavior of potassium magnesium phosphate cement (PMPC) paste, the sulfate content, strength, and length of PMPC specimens were measured at different corrosion ages under 5% Na_2_SO_4_ solution soaking conditions, and the phase composition and microstructure were analyzed. The conclusion is as follows: In PMPC specimens subjected to one-dimensional SO_4_^2−^ corrosion, the relation between the diffusion depth of SO_4_^2−^ (*h*) and the SO_4_^2−^ concentration (*c* (*h*, *t*)) can be referred by a polynomial very well. The sulfate diffusion coefficient (*D*) of PMPC specimens was one order of magnitude lower than Portland cement concrete (on the order of 10^−7^ mm^2^/s). The surface SO_4_^2−^ concentration *c* (0, *t*), the SO_4_^2−^ computed corrosion depth *h*_00_, and *D* of FM2 specimen containing 20% fly ash (FA) were all less than those of the FM0 specimen (reference). At 360-day immersion ages, the *c* (0, 360 d) and *h*_00_ in FM2 were obviously smaller than those in FM0, and the *D* of FM2 was 64.2% of FM0. The strengths of FM2 specimens soaked for 2 days (the benchmark strength) were greater than those of FM0 specimens. At 360-day immersion ages, the residual flexural/compressive strength ratios (360-day strength/benchmark strength) of FM0 and FM2 specimens were all larger than 95%. The volume linear expansion rates (*S_n_*) of PMPC specimens continued to increase with the immersion age, and *S_n_* of FM2 specimen was only 49.5% of that of the FM0 specimen at 360-day immersion ages. The results provide an experimental basis for the application of PMPC-based materials.

## 1. Introduction

For concrete structures located in seawater, saline-alkali land, salt lakes, and other environments rich in SO_4_^2−^, SO_4_^2−^ can infiltrate the concrete structure through the initial imperfection of the concrete. Expansive products containing SO_4_^2−^ can be generated through the reactions between the hydration products and SO_4_^2−^, resulting in the pore structure degradation, through which the SO_4_^2−^ content of the environment can quickly infiltrate the structure and cause the further deterioration of the concrete properties [1,2,3]. The porous properties and instability of hydration products in Portland cement paste render it unsuitable in environments with relatively high SO_4_^2−^ contents [1,2].

The hydration product of magnesium phosphate cement (MPC) pastes, namely phosphate hydrate, is strongly ionic bonded and has a dense structure. As a result, it can effectively hinder the penetration of harmful ions [4,5,6,7]. At present, the most researched and applied types are ammonium magnesium phosphate cement (AMPC) composed of dead burned MgO powders and NH_4_H_2_PO_4_, and potassium magnesium phosphate cement (PMPC) composed of dead burned MgO powders and KH_2_PO_4_ (KDP). Among them, PMPC has received more attention by reason of its relatively adjustable setting time and non-polluting construction process to the environment. The performance of MgNH_4_PO_4_·6H_2_O and MgKPO_4_·6H_2_O is stable when the pH value = 7–11 and they have high salt corrosion resistance [4,5,6,7]. Therefore, the application of MPC-based materials in the fields of structural repair [8,9] and anti-corrosion [10,11,12] has been extensively studied in the literature. The incorporation of some industrial solid waste powders can reduce the cost of MPC and improve its properties [4,5,6,7]. Among the various industrial solid waste powders, low calcium fly ash (FA) has a better modification effect, which can improve the flowability of fresh MPC paste [13], the later strength, the volume stability, the water stability, as well as the acid and salt corrosion resistance of hardened MPC paste [14,15,16,17].

Through measuring the strength change, mass loss, and length change of PMPC test pieces subjected to different solution freeze–thaw cycles, L. Chong et al. [17] proved that the degree of structural degradation of the specimens is obviously related to the type of corrosive medium. By analyzing the development of strength and deformation of PMPC test pieces soaked in water and a 5% Na_2_SO_4_ solution, it can be determined that adding an appropriate amount of FA retards the structure degradation of hardened MPC paste [18]. Through measuring the strength change of the specimens in solutions, references [19] and [20] suggested that the order of the influence of corrosive media on the degree of strength change of PMPC specimens was as follows: 5% Na_2_SO_4_ solution is the most severe, followed by water, and it is the lightest in 3.5–5% NaCl solution. The mass change, strength change, and strain of cement-based material test pieces are commonly used in lab test to evaluate the resistance of cement-based materials to sulfate attack. These parameters cannot reflect the transport of SO_4_^2−^ inside the hardened cement-based materials [21,22].

During the entire process of sulfate corrosion leading to the failure of the hardened structure of cement concrete, the diffusion of SO_4_^2−^ in a hardened structure is a prerequisite for the occurrence of corrosion action and damage [21], the newly defined integral area of SO_4_^2−^ distributions is considered an appropriate indicator to describe the deterioration law of concrete subjected to sulfate attack [22]. A deep understanding of the transport law and the diffusion mechanism of SO_4_^2−^ inside the MPC system is crucial for further revealing the sulfate corrosion mechanism in MPC-based materials and for fabricating robust MPC, in terms of corrosion resistance. The sulfate corrosion action of a PMPC system with steel slag powders was systematically investigated by our team. More specifically, the content distribution of sulfate ions in PMPC paste11 specimens with some steel slag powders (SSP) immersed in 5% Na_2_SO_4_ solution for a long time was determined and analyzed. The results confirmed that a moderate amount of SSP can reduce the SO_4_^2−^ diffusion coefficient of PMPC paste11 specimens. However, the improvement effect was limited [23]. The existing research [13,14,15,16,17] has confirmed that low-calcium FA has a good modification effect on the macroscopic physical mechanical properties of the MPC system. However, the study on the diffusion law of sulfate in the FA-MPC system has not been conducted yet. In the study, the transport law and corrosion behavior of an FA-PMPC paste under 5% Na_2_SO_4_ solution soaking conditions were explored by measuring the sulfate content, strength, and length of PMPC specimens in different corrosion ages. The study results will provide an experimental basis for the application of PMPC-based materials.

## 2. Experiments

### 2.1. Materials

**Figure 1:**
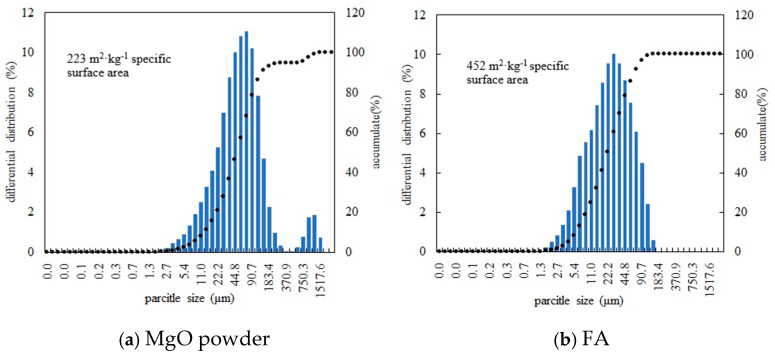
The parameters related to fineness of MgO powder and FA.

The dead burnt MgO powder was offered by the Haicheng Guangda High Purity Magnesia Co., Ltd. in Haicheng, Liaoning Province, China. The MgO powder is obtained by electric melting of magnesite at a temperature higher than 1500 °C and then grinding it into powder. The specific surface area and the particle size distribution of the MgO powders were measured by a LS-609 Laser Particle Size Analyzer (made in Oumoke Instrument Co., Ltd. in Zhuhai, Guangdong Province, China; Work environment: 20 °C ± 3 °C, Relative humidity ≤ 85%); the measuring results are shown in Figure 1a. The particle size of D10 (the particle size corresponding to the cumulative particle size distribution percentage of the sample reaching 10%), D50, and D90 is 12.62 μm, 48.75 μm, and 138.05 μm, respectively. The used FA (class F) came from the Zhenjiang Jianbi Power Plant in Zhenjiang, Jiangsu Province, China. The specific surface area and the laser particle size distribution of FA are shown in Figure 1b, and the particle size of D10, D50, and D90 is 5.87 μm, 21.88 μm and 65.20 μm, respectively. Table 1 presents the composition of MgO powder and FA obtained from XRF analysis (Q4 TASMAN Spectrometer, made by the Metek Group—Germany Spike Analytical Instruments Company, Elmshorn, Germany).

### 2.2. Mix Proportions of PMPC Paste and Specimen Preparation

Referring to the existing research results [17,18], Table 2 lists the mix proportions of the PMPC paste. According to the result of Table 2, the water/binder ratio (W/B) of the freshly mixed FM1 and FM2 paste with 10–20% FA was less than the W/B of the FM0 paste without FA when the fluidity of the paste was basically the same.

By maintaining an ambient temperature of 20 ± 5 °C and a relative humidity of 50% to 70%, the mass of acid component, base component, retarder, and water was calculated and weighed in accordance with Table 2. CR, KDP, and water were first poured into the mixing pot of the cement sand mixer and slowly stirred for 60 s. MgO powder and FA were slowly added and slowly stirred for 60 s. When the stirring stopped, the paste on the blades scraped off. After performing rapid stirring for 120 s, a PMPC paste was obtained. Referred to Chinese Standard GB/T2419-2005 [24], the fluidity of the freshly mixed paste was measured. The different sizes of the paste test pieces were made, namely a Φ 5 cm × 10 cm cylinder (for SO_4_^2−^ content), a 4 cm × 4 cm × 16 cm prism (for strength), and a 2.5 cm × 2.5 cm × 28 cm prism (for deformation). The test piece is demolded after 5 h (except for the Φ 5 cm × 10 cm cylinder). The test pieces were placed in the curing room at 18–22 °C and 50–80% RH until 28 days (672 h).

### 2.3. Test Methods

#### 2.3.1. Exposure of Specimens

**Figure 2:**
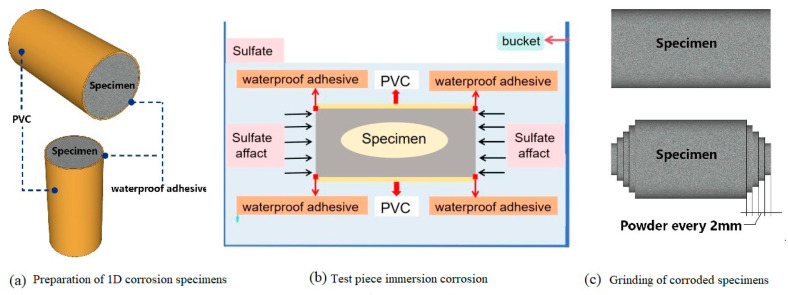
Preparation of one-dimensional sulfate corrosion specimens and sample powder.

With respect to existing research [25,26,27], 5% Na_2_SO_4_ (mass fraction) solution was used as a corrosive medium for the sulfate fill soaking test of the PMPC test pieces. When the hydration age reached 28 days, the reference FM0 and FM2 (with 20% FA) were taken for corrosion testing. For each composition, four sets of specimens were soaked for sulfate content measurement (2 pieces per set), four sets of specimens were soaked for strength measurement (3 pieces per set), and one set of specimens were soaked for deformation measurement (3 pieces per set). The cylindrical test pieces were sealed with waterproof adhesive before exposure test for meeting the one-dimensional transmission of SO_4_^2−^ (Figure 2a,b). The volume ratio of the sulfate solution to the specimens was larger than 5. The corrosive solution was replaced every 15 days to ensure that the Na_2_SO_4_ concentration in the solution reached 5% (mass fraction).

#### 2.3.2. Measurement of Sulfate Concentration

When attaining the set corrosion age, some Φ 5 cm × 10 cm test pieces were taken out and cleaned. The test pieces were dried to a constant mass at below 60 °C, the test pieces were then cut and ground into powder along the opposite end face (Figure 2c), ensuring that the particle size of the powder is less than 200 mesh. According to GB/T 5750.5-2023 [28], GB/T 749-2008 [26], and existing research [23], the SO_4_^2−^ content was measured in the powders with turbidimetric method.

#### 2.3.3. Fluidity, Strength and Deformation Measurement

The fluidity of fresh PMPC paste was measured referring to GB/T2419-2005 [24] and is presented in Table 2. The strength of PMPC specimens was measured according to GB/T 17671-2021 [29]. At an ambient temperature of 20 ± 5 °C, a universal testing machine (WED-300, Manufactured by Wuxi Jianyi Co., Ltd., Wuxi, Jiangsu, China) was used to measure the flexural strength (1 group of 3 pieces) and compressive strength (1 group of 6 pieces) of the specimens, where the loading speed for flexural load is 50 N/s ± 10 N/s and the loading speed for compressive load is 2400 N/s ± 200 N/s. The mechanical results at different ages are presented in Table 2. Referring to JC/T 603-2004 [30], the sizes of the test pieces were measured, and the length change rate (*S_n_*) of the test piece can be calculated as follows:

*S_n_* = (*L*_0_ − *L_t_*) × 100%/250 (1)

where *L*_0_ and *L_t_* (mm) are the lengths of the saturated surface dry specimens immersed for 48 h and t days, separately; 250 mm is the effective length of the specimens.

#### 2.3.4. Microstructural Analysis

Small sample pieces were taken for microanalysis (containing skin, thickness: 5–10 mm). The sample pieces were initially cleaned, and some of them were ground into powder (the grain size is less than 200 mesh). The samples for microanalysis were immersed in isopropanol. The powder sample and the small sample pieces were dried to a constant weight (50–60 °C) before the analysis. The morphology and the elemental compositions of samples were observed and analyzed by using a field emission scanning electron microscope (SEM) and an X-ray energy spectrum analyzer (EDS). The phase composition of the sample was analyzed by conducting X-ray diffraction analyzer (XRD) and employing a thermal analyzer (TG and DTG). The pore structure of the sample was analyzed by using a fully automatic porosity analyzer (MIP). The project, instrument, and analysis conditions for the microanalysis of samples are listed in Table 3.

## 3. Results and Analysis

### 3.1. Microstructure Characteristics and Products

SEM-EDS, MIP, XRD, and TG is a commonly used method for analyzing the microstructure and phase composition of PMPC paste. In reference [31], the white precipitate on the surface of PMPC paste specimens immersed in water for 240 days were analyzed by XRD, and the result showed that, in addition to MgKPO_4_·6H_2_O, a new product Mg_3_(PO_4_)_2_·22H_2_O was present on the surface of the specimens. This verified that MgKPO_4_·6H_2_O partially dissolves and hydrolyzes to form new hydration products under long-term water immersion, with the corresponding chemical reaction shown in Equation (1). SEM analysis of the eroded surface of the PMPC sample revealed that originally an intact structure had disintegrated into numerous individual units with flaking at the ends. A large number of needle-like and rod-shaped crystals were randomly reconnected around the MgKPO_4_·6H_2_O prism ends, which is the result of ion recrystallization to form new hydration products after the dissolution of MgKPO_4_·6H_2_O. These crystals have poor crystallinity and loose connections, with many small blocky crystals distributed on their surfaces.

3MgKPO_4_·6H_2_O(K-Struvite) + 4H_2_O → Mg_3_(PO_4_)_2_·22H_2_O(cattiite) + K_3_PO_4_

Liming Lv [16] confirmed through MIP analysis that incorporating 30% FA can significantly improve the pore structure of PMPC paste after 28 days of hydration, and the degree of deterioration of PMPC paste’s pore structure after 152 days of water immersion is significantly reduced. Based on the analysis results of XRD and SEM, it is inferred that the filling effect and active effect of FA enhance the overall density of PMPC paste, which may improve its durability. In reference [20], the XRD analysis of the PMPC sample (immersed in 5% sodium sulfate solution for 180 days) confirmed that the sulfate crystal MgSO_4_⋅7H_2_O was detected, which indicates that SO_4_^2−^ in the 5% Na_2_SO_4_ solution can combine with some cations in the PMPC paste to generate MgSO_4_·7H_2_O. In reference [32,33], the XRD and TG-DTG analysis confirmed that there were Mg (OH)_2_ and MgCO_3_ in the PMPC sample (immersed in water and 5% Na_2_SO_4_ for over 360 days). It is inferred that in a long-term water-saturated environment, unreacted MgO will partially slake into Mg (OH)_2_, causing the pH of the soaking solution to continuously increase and develop towards an alkaline environment. Carbon dioxide from the air will dissolve into the soaking solution and participate in the reaction, generating MgCO_3_.

#### 3.1.1. XRD Analysis

**Figure 3:**
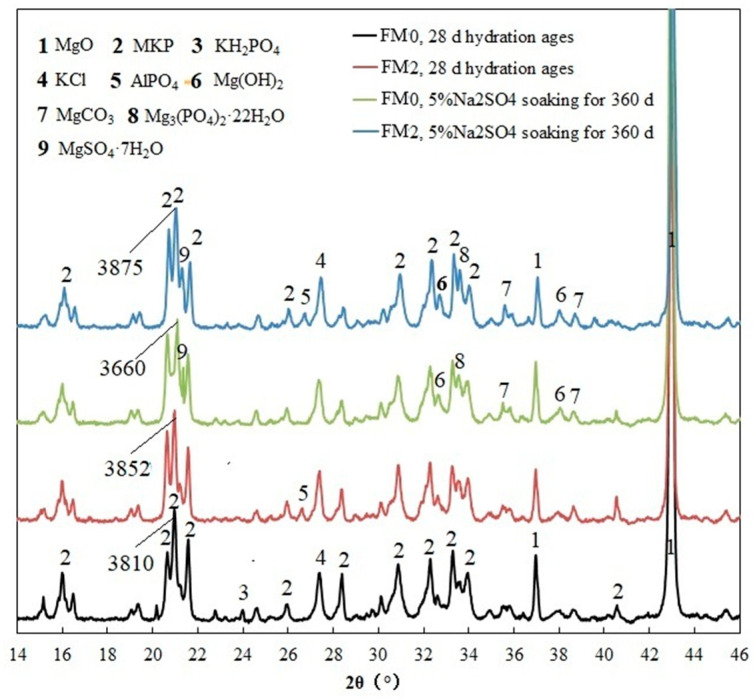
XRD patterns of PMPC samples.

XRD analysis was performed on the PMPC samples that had been hydrated for 28 days and fully immersed in sulfate for 360 days. As can be seen in Figure 3, many diffraction peaks of MgKPO_4_·6H_2_O (PDF#01-072-1605) can be detected. In particular, two diffraction peaks of unreacted MgO at 2θ = 36.7–37.2° and 2θ = 42.6–43.6° (PDF#45-0946), and a diffraction peak of KCl crystal at 2θ ≈ 27.0–27.7° (PDF#00-006-0478) for four kinds of samples can be observed. The KCl crystal was generated by combining Cl^−^ with K^+^ in the paste. In the FM0 sample that has been hydrated for 28 days, the diffraction peak of KH_2_PO_4_ at 2θ ≈ 24.0° (PDF#00-001-0894) suggests that there is still unreacted KDP in the FM0 sample that has been hydrated for 28 days. The same characteristic peak was not observed in the FM2 sample that has been hydrated for 28 days, indicating a more complete hydration reaction of the FM2 paste. Nevertheless, the characteristic peak of AlPO_4_ (2θ = 27.0–27.6°, PDF#01-076-0230) was added in the FM2 sample after 28 days of hydration. This peak should originate from the reaction of the active Al_2_O_3_ in the raw material FA of PMPC with phosphate. The analysis results are consistent with previous research [15,16], confirming that FA can promote hydration (dispersion and heterogeneous nucleation) and has activity in PMPC system.

The FM0 and FM2 samples subjected to 360-day sulfate corrosion exhibited the stronger characteristic peaks of Mg (OH)_2_ than the FM0 and FM2 samples hydrated for 28 days at 2θ ≈ 32.8° and 38.1° (PDF#01-075-1527), confirming the slaking of exposed MgO. Two characteristic peaks of MgCO_3_ (2θ ≈ 35.6° and 38.8°, PDF#96-900-1854) were also observed in the FM0 and FM2 samples with 360-day soaking ages, indicating the carbonation of Mg (OH)_2_. There is a characteristic peak of Mg_3_(PO_4_)_2_·22H_2_O at approximately 2θ = 33.52° (PDF#00-035-0186). The analysis results are consistent with previous research [31]. The FM0 and FM2 samples subjected to sulfate corrosion exhibited the characteristic peaks of MgSO_4_·7H_2_O at 2θ = 21.3–21.5° (PDF#00-036-0419), confirming that the SO_4_^2−^ diffusing into the pore solution of the hardened body will combine with hydrolyzed Mg^2+^ to form MgSO_4_·7H_2_O crystals. The analysis results are consistent with previous research [32,33].

Compared to the FM0 samples that were hydrated for 28 days and soaked for 360 days in Figure 3, significant differences between the peak intensities of the main characteristic peak of MgKPO_4_·6H_2_O at 2θ ≈ 21° (3810 and 3660) can be observed. The lower peak intensity (3660) of the FM0 sample with 360 soaking ages verified the reduction of MKP content in the sample. At 2θ ≈ 21°, there are basically equal characteristic peak intensities of MgKPO_4_·6H_2_O (3852 and 3875) in two kinds of FM2 samples (28-day hydration ages and 360 soaking ages). XRD analysis results proved that the loss of MgKPO_4_·6H_2_O in FM2 samples caused by long-term salt solution corrosion is relatively small, which are consistent with previous research [20,31].

#### 3.1.2. SEM-EDS Analysis

**Figure 4:**
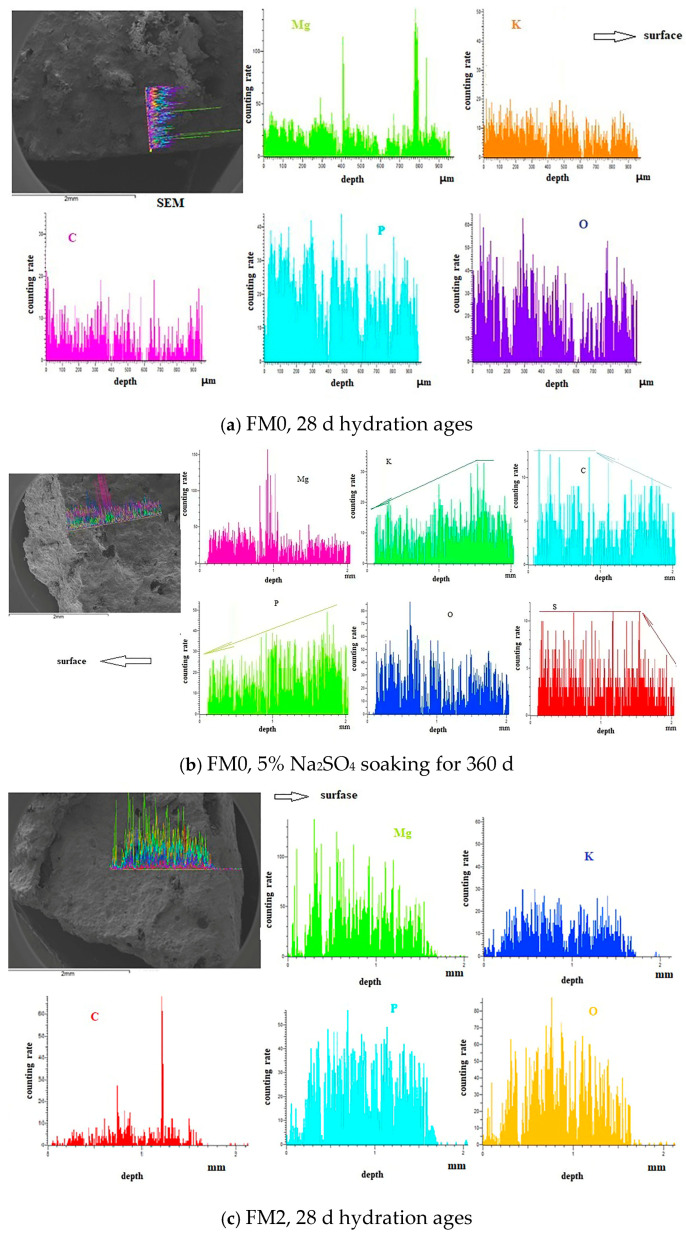

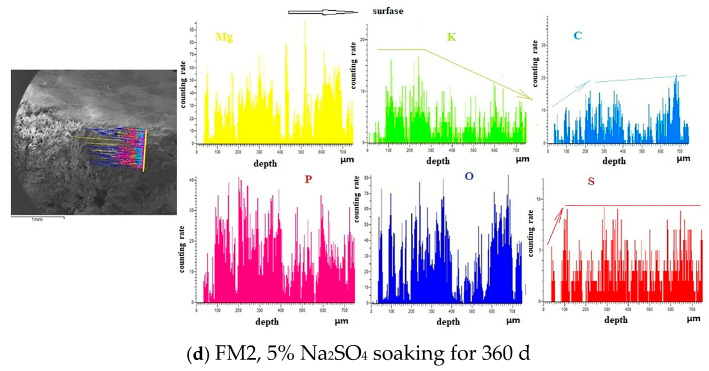
SEM line scan results of PMPC samples.

By scanning a designated line of a PMPC paste sample using a field emission SEM, the variation curve of the element of interest content on this line can be obtained. Figure 4 shows the distribution of each element content obtained by scanning vertically from the surface to the interior of the paste sample. The variation in the height of the same element along a straight line is mainly caused by changes in element content, fluctuations in X-ray counting statistics, and geometric factors. Due to the scanning line segment length not exceeding 2 mm, the changes in the geometric factors on the sample surface can be ignored, and the height of the distribution curve can approximately represent the element content.

Figure 4a shows the line scan element distribution of the FM0 sample that has been hydrated for 28 days. The Mg element content is basically similar in the scanning range except for a sharp increase at 2 points (which should be caused by unreacted magnesium oxide particles). The online scanning range for the content of K, P, O, and C elements is basically similar. A certain amount of carbon element confirms the carbonation phenomenon on the surface of the PMPC sample; it is consistent with the XRD result in Figure 3. Figure 4b shows the line scan element distribution of the FM0 sample soaking for 360 d in 5% Na_2_SO_4_ solution. The sharp increase in the Mg and O content at point 1 is caused by unreacted magnesium oxide particles. At a distance of about 1.5 mm and 1.7 mm from the surface, the content of K and P elements gradually decreases as it approaches the surface, which suggests a dissociation of K ions and PO_4_^3−^ in MKP near the surface. The content of C and S was significantly higher near the surface (within a depth of 1.1 mm and 1.5 mm from the surface) but gradually decreased as it extended inward. The high content of C and S on the surface indicates the presence of carbonation and sulfate penetration phenomena, which is consistent with the XRD result in Figure 3. However, as they extend into the interior of the PMPC sample, the carbonation and sulfate penetration phenomena gradually decrease.

Figure 4c shows the line scan element distribution of the FM2 sample with 28-days hydration ages. The online scanning range for the content of Mg, K, P, and O elements was basically similar. A certain amount of carbon element confirms the carbonization phenomenon on the surface of the PMPC sample; it is consistent with the XRD result in Figure 3. The sharp increase in the C content at point 2 was caused by the conductive adhesive. Figure 4d shows the line scan element distribution of the FM2 sample soaking for 360 d in 5% Na_2_SO_4_ solution. The online scanning range for the content of Mg, P, and O elements was basically similar. At a distance of about 500 μm from the surface (obviously lower than that in Figure 4b), the content of K element gradually decreased as it approached the surface. This outcome suggests the dissociation of K^+^ in MgKPO_4_·6H_2_O near the surface. The presence of a small amount of C and S elements confirms the carbonation and sulfate penetration on the surface of the PMPC sample, and the distribution width (distance from the surface, about 500 μm and 700 μm) of the C and S elements in the sample FM2 was significantly less than that in the FM0 sample (Figure 4b).

**Figure 5:**
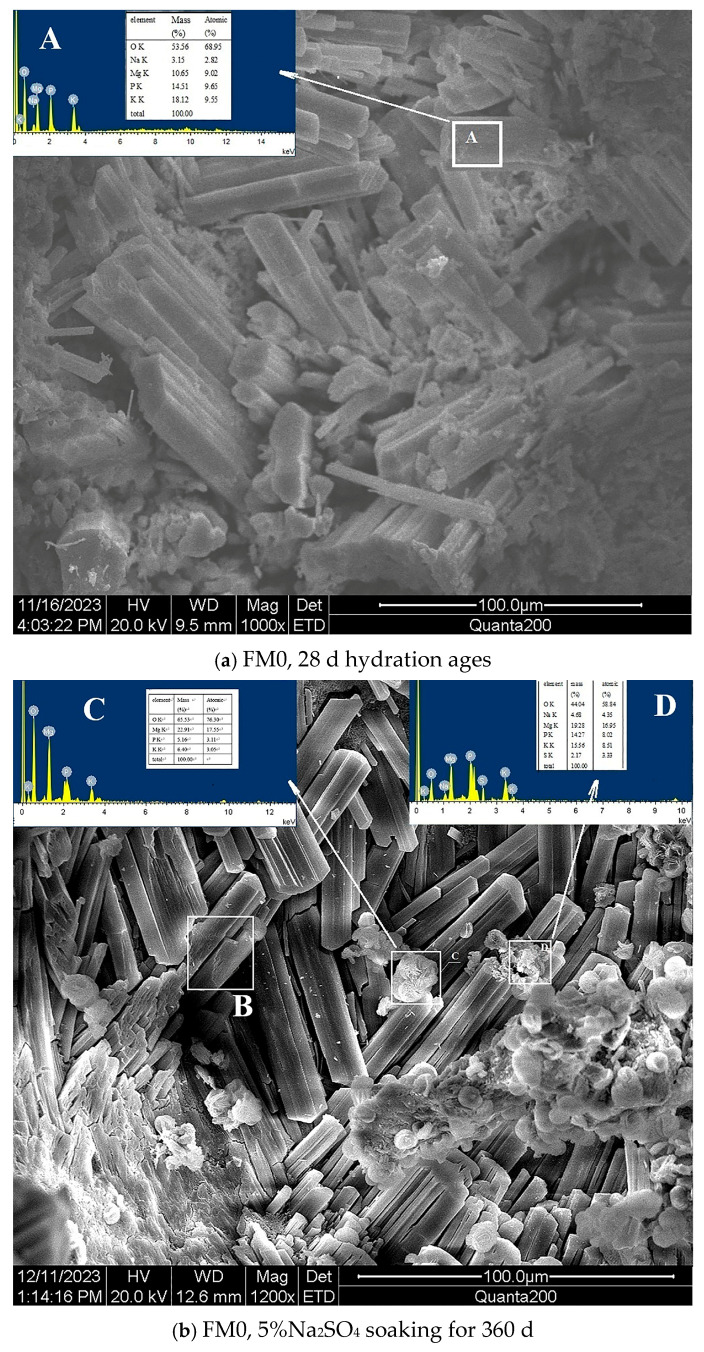

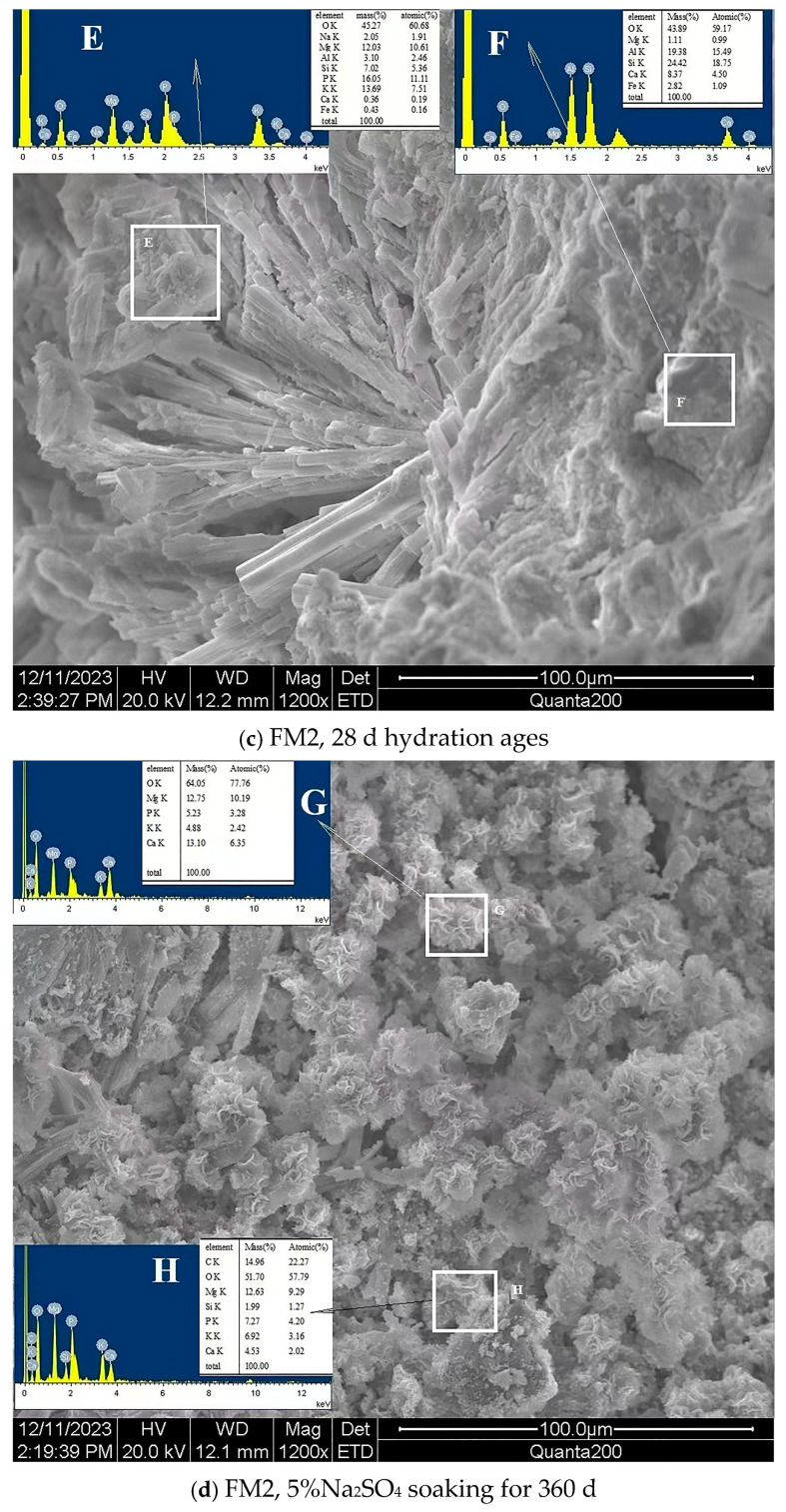
SEM-EDS image of PMPC sample.

In Figure 5a, the columnar crystals in pores of the FM0 sample that has been hydrated for 28 days were loosely accumulated. There were obvious corrosion marks in the smooth crystal surface. The area A in Figure 5a was composed of O, Mg, P, K, and Na. The m_Mg_:m_P_:m_K_ was 9.02:9.65:9.55 (Table 4), the columnar crystal was speculated to be MgKPO_4_·6H_2_O combined with Figure 3. In the FM0 sample with 360-day corrosion ages, there were numerous defects on the surface of the columnar crystal (for example area B in Figure 5b). Some gel phase or crystals with low crystallinity were adhered to the surface of the MgKPO_4_·6H_2_O crystal or stacked between the MgKPO_4_·6H_2_O crystals. The m_Mg_:m_P_:m_K_ was 17.55:3.11:3.05 in area C (Table 4), which should be recrystallized MgKPO_4_·6H_2_O and Mg (OH)_2_ combined with Figure 3 and reference [31,32,33]. The area D was made up of O, Mg, P, K, Na, and S (Table 4), which was speculated to be MgKPO_4_·6H_2_O and MgSO_4_·7H_2_O combined with Figure 3 and reference [20].

Compared with the FM0 sample in Figure 5a, the accumulation of the crystals in pores of the FM2 sample with 28-day hydration ages was more compact in Figure 5c. The area E in Figure 5c was composed of Mg, P, K, Na, Al, Si, Na, Ca, Fe, and O (Table 4), and m_Mg_: m_P_ was close to 1, suggesting that the amorphous phase should be MgKPO_4_·6H_2_O. The morphology of MgKPO_4_·6H_2_O is affected by the presence of Ca, Fe, Al, and Si element inclusions. The area F was composed of Al, Si, Ca, Fe, Mg, and O, and the glass body should be fly ash.

#### 3.1.3. TG-DTG Analysis

**Figure 6:**
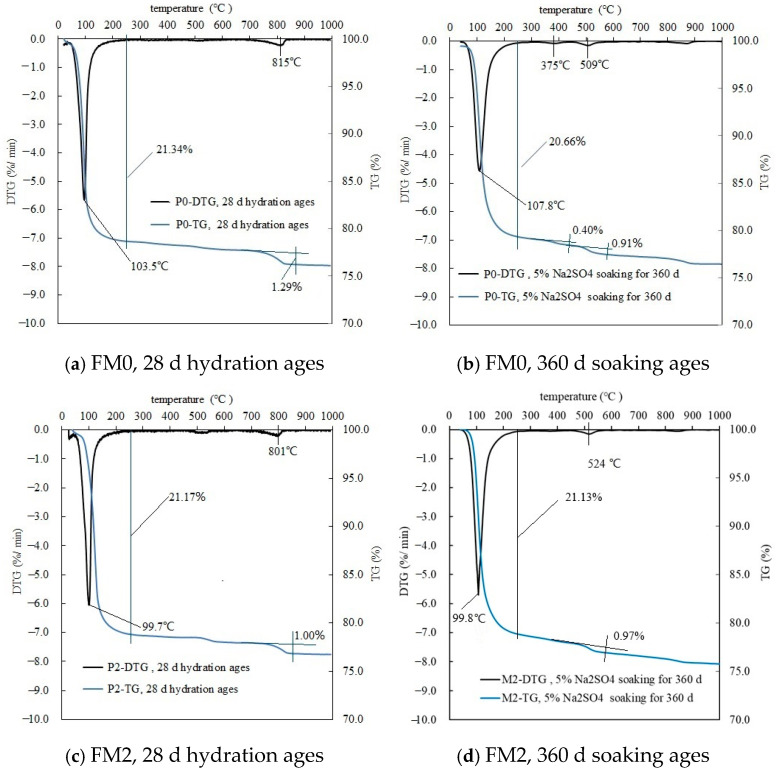
TG-DTG of PMPC samples.

As can be seen in Figure 6, because MgKPO_4_·6H_2_O in the PMPC samples lost crystallization water at about 100 °C [4,5,6,7], there is a mass loss on the TG curve (around 100 °C). The FM0 sample with 360-day corrosion ages (20.66%, Figure 6b) showed less mass loss compared to the FM0 sample with 28 days of hydration age (21.34%, Figure 6a), suggesting the dissolution of MgKPO_4_·6H_2_O in the FM0 sample with 360-day corrosion ages. Additionally, the FM0 sample with 360-day corrosion ages exhibited an additional mass loss of 0.40% with a maximum decomposition rate at about 375 °C and an additional mass loss of 0.91% with a maximum decomposition rate at about 509 °C, respectively. Combined with the analysis results in Figure 3 and existing research [31,32,33], it is suggested that the thermal decomposition of Mg (OH)_2_, and MgCO_3_ led to the formation of the mass loss and the heat valley.

In the FM2 sample with 28-day hydration ages in Figure 6c, a mass loss (21.17%) at 60–200 °C was observed, which was slightly lower than the mass loss (21.34%) of the FM0 sample in Figure 6a and was attributed to the decrease in the content of the MgO powders. In Figure 6d, the mass loss of the FM2 sample with 360-day corrosion ages at 60–200 °C (21.13%) was almost the same as the mass loss of the FM2 sample with 28-day hydration ages (Figure 6c, 21.17%), suggesting an obvious reduction in the dissolution of MgKPO_4_·6H_2_O in the FM2 sample with 360-day corrosion ages. The FM2 sample with 360-day corrosion ages showed one obvious mass loss (0.97%) with a maximum decomposition rate at about 524 °C. Combined with the analysis results in Figure 3 and existing research [31,32,33], the decomposition of MgCO_3_ led to the formation of the mass loss and the heat valley.

#### 3.1.4. MIP Analysis

**Figure 7:**
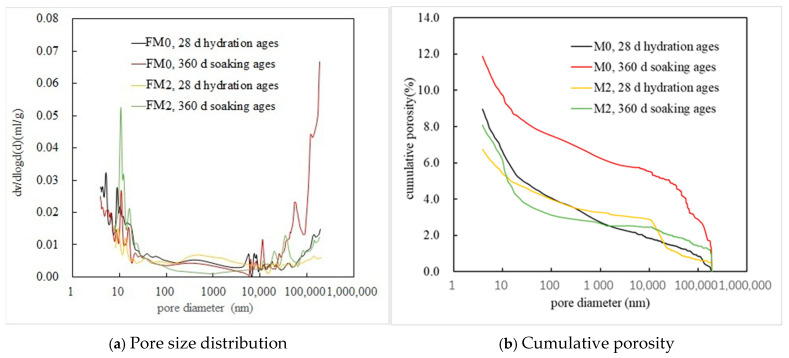
MIP results of PMPC samples.

Figure 7 and Table 5 list the MIP analysis results of the PMPC samples. Compared to the FM0 sample that had been hydrated for 28 days (Figure 7b and Table 5), the FM0 sample with 360-day corrosion ages had a larger total porosity and ratio of harmful pores (≥200 nm) (12.52% and 62.17%), suggesting that sulfate immersion corrosion led to deterioration of the pore structure of PMPC paste, which is consistent with previous research [16,31]. The FM2 sample that had been hydrated for 28 days had a smaller total porosity (6.75%) compared to FM0 that had been hydrated for 28 days, suggesting that some FA can improve the compactness of the hardened PMPC paste, which is consistent with previous research [15,16]. In Figure 7b and Table 5, compared to the FM2 that had been hydrated for 28 days, the FM2 sample with 360-day soaking ages had a larger total porosity (8.10%), more content of harmless pores (<50 nm, 57.54%), but a smaller proportion of multiple harmful pores (36.37%). The results of the pore structure parameters suggest that sulfate immersion corrosion could lead to degradation of the pore structure in an FM2 specimen, which is consistent with previous research [16]. However, the degree of deterioration of the pore structure was obviously decreased compared to the FM0 specimens. Additionally, some newly generated phases during the sulfate immersion corrosion process (for example, MgSO_4_·7H_2_O) can fill the capillary pores of the PMPC paste and refine its pore size distribution.

### 3.2. SO_4_^2−^ Content Test and Analysis Inside PMPC Paste

#### 3.2.1. Diffusion Rule of SO_4_^2−^ Inside PMPC Paste

**Figure 8:**
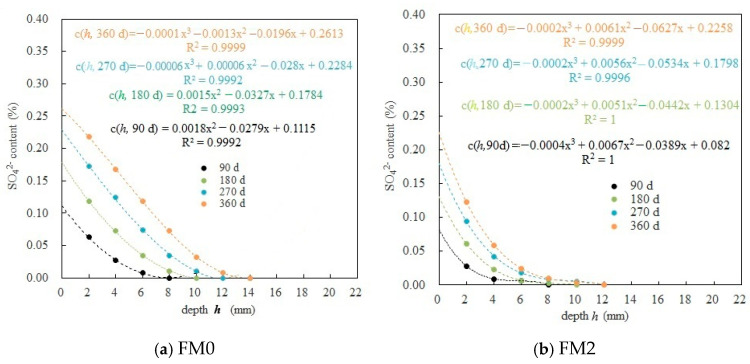
Distribution of SO_4_^2−^ concentration in FM0 and FM2 specimens.

Figure 8 reflects the distribution of SO_4_^2−^ concentration (*c* (*h*, *t*)) inside the PMPC specimen along the diffusion depth of SO_4_^2−^ (*h*). The polynomial formulas fitted to *c* (*h*, *t*) with *h* in the PMPC specimens at 90 d, 180 d, 270 d, and 360 d (soaking ages *t*) are listed in Figure 8. The correlation coefficients of the polynomial models were greater than 0.999. The SO_4_^2−^ content (*c* (*h*, *t*)) at the same corrosion depth (*h*), as well as the penetration depth of SO_4_^2−^ (*h*_0_, depth measured when the SO_4_^2−^ content is 0) gradually increased with corrosion age (*t*). Under the same conditions, the *c* (*h*, *t*) in the FM2 specimen was lower than the *c* (*h*, *t*) of the FM0 specimen, while the *h*_0_ of the FM2 specimen was less than the *h*_0_ of the FM0 specimen. During the 360-day soaking period, the *h*_0_ of the FM2 specimen (≤12 mm) was significantly lower than the *h*_0_ of the FM0 specimens (≤14 mm). The results suggest that a reasonable amount of FA will obviously improve the sulfate penetration resistance of hardened PMPC paste, which is consistent with the conclusion of the existing research results in the literature [15,16,18,19].

#### 3.2.2. Calculation of Fitting Parameters for SO_4_^2−^ Diffusion in PMPC Paste

According to the literature [1,2,3], Fick’s second law (Formula (2)) was used to describe the diffusion law of SO_4_^2−^ in cement-based materials:

(2) $$\frac{\partial c}{\partial t}=D\frac{\partial c}{\partial x^{2}}$$

According to boundary conditions of references [33], the Laplace transform, and its inverse, the analytical solution of Equation (2) can be obtained as follows:

(3) $$c(x,t)=c_{s}-(c_{s}-c_{0})erf(x/2\sqrt{Dt})$$

of which $erf(x/2\sqrt{Dt})$ is the error function.

According to reference [33], the polynomial formulas in Figure 8 were used for calculating corrosion depth *h*_00_ of FM0 and FM2 test pieces with different corrosion ages (*t*) and then calculating *c_s_* (0, *t*) (h = 0) and *c* (*h*_00_, *t*) (*h* = *h*_00_). Substituting *c_s_* (0, *t*), *c* (*h*_00_, *t*) and *c*_0_ = 0 into Equation (3), the $erf(x/2\sqrt{Dt})$ value was computed (Table 6). $x/2\sqrt{Dt}$ can be found in the Gaussian error function table, and the *D* (diffusion coefficient) can be computed. The relevant parameters are listed in Table 6.

The results in Table 6 indicate that *c_s_* (0, *t*) gradually grows with *t*. The *c_s_* (0, *t*) of the FM2 test pieces was obviously less than that of the FM0 test pieces. At 360-day corrosion ages, *c_s_* (0, 360 d) of FM2 (0.2258%) was only 86% of that of FM0 (0.2613%). At 90-day corrosion ages, the *h*_00_ (7 mm) of the FM2 test piece was less than the *h*_00_ of the FM0 test piece (8 mm). At 360-day corrosion ages, the *h*_00_ in the FM2 test piece (11 mm) was obviously smaller than the *h*_00_ of FM0 test piece (14 mm). The *D* values in the PMPC test pieces at all soaking ages were all on about 10^−7^ mm^2^/s, less than that of the Portland cement concrete in the previously reported studies (on about 10^−6^ mm^2^/s) [1,2,3]. At 90-day and 180-day corrosion ages, the *D* showed a downward trend. At 270-day and 360-day corrosion ages, the *D* gradually increased. The *D* of FM2 test pieces was always smaller than that of the FM0 test pieces under the same conditions. At 90 d, the *D* of the FM2 test piece was 64.2% of the *D* of the FM0 test piece. At 360 d, the *D* of the FM2 test piece was only 74.4% of the FM0 test piece. The results above suggest that the PMPC paste has a strong resistance to SO_4_^2−^ diffusion (consistent with the conclusion of existing research results [23]), and some FA will significantly enhance the SO_4_^2−^ diffusion resistance and stability of the PMPC paste under a sulfate corrosion environment [15,16,18,19].

### 3.3. The Mechanical Properties of PMPC Specimens

#### 3.3.1. Flexural and Compressive Strength

The length change and strength change can be used as a macroscopic indicator of the resistance of cement concrete to sulfate corrosion [25,26]. Table 7 reflects the change in the flexural strength (FS) and compressive strength (CS) of PMPC specimens with immersion ages. Based on the FS and CS of the specimens soaked for 2 days as the benchmark strength, the FM2 containing FA had larger FS and CS, compared to the reference FM0. The FS and CS of the PMPC specimens first rose and then fell with the corrosion age, and the peak value was reached at 180-day immersion ages. At 360-day immersion ages, the residual flexural/compressive strength ratios (360-day strength)/benchmark strength of the FM0 and FM2 test pieces ((95.6%/99.2% and 96.7%/98.3%) were all larger than 95%). The strength change amplitude for FM2 was obviously smaller than that of the FM0, and their residual strength rates were slightly greater than that of FM0. Therefore, some FA can decrease the strength degradation of PMPC paste, which is in direct line with the existing research results [15,16,18,19].

#### 3.3.2. Deformation

**Figure 9:**
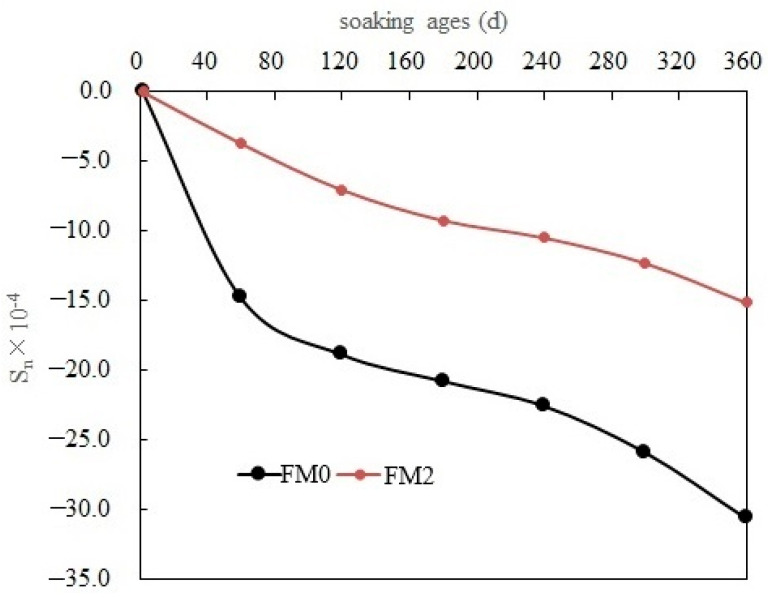
*S_n_* of PMPC test pieces with different SO_4_^2−^ corrosion ages.

Figure 9 shows the length change rate (*S_n_*) of PMPC test pieces with different SO_4_^2−^ corrosion ages. According to formula (1), if *S_n_* is negative, it can be argued that the specimen has undergone expansion. The volume linear expansion rates (*S_n_*) of the FM0 and FM2 specimens continued to rise with the immersion age. The growth rate of *S_n_* was fast during the 120-day soaking periods, slowed down during the 120–240-day soaking periods and accelerated after the 240-day soaking periods. At 360-day immersing ages, the *S_n_* of the FM0 and FM2 specimens were −30.61 × 10^−4^ and −15.14 × 10^−4^, respectively. The volume linear expansion rate of the FA-modified FM2 specimen was only 49.5% of that of the reference specimen FM0. Therefore, some FA can decrease the expansion of hardened PMPC paste, which is consistent with the conclusions of the existing research results [18].

## 4. Discussion

Under the condition of full immersion in the solution, the initial SO_4_^2−^ permeation into PMPC specimens depends on a SO_4_^2−^ content gradient from the surface to the interior of the specimens and the initial pore structure of the PMPC specimens [1,2,3]. Change in SO_4_^2−^ diffusion depends on changes in the pore structure of PMPC specimens [1,2,3,23]. The initial strength of the PMPC specimens depends on the amount of generated MgKPO_4_·6H_2_O, the distribution of the central matrix with higher strength (example for unhydrated MgO particles and inert powder filler) on the surrounding base phase, and the pore structure [18,23]. A change in the strength of the PMPC specimens can be dependent on the stability of MgKPO_4_·6H_2_O and changes in the pore structure [18,23]. The deformation of a PMPC specimen hinges on the type and amount of expansive phase generated in the PMPC paste [18]. Combining the existing research findings [1,2,3,16,18,23,31,32,33] with the results of this work, the sulfate corrosion behavior in PMPC paste can be explained as follows:

a. The SO_4_^2−^ in the hardened PMPC paste becomes enriched in shallow areas close to the surface due to the lower initial porosity. The low W/B leads to the presence of unreacted KH_2_PO_4_ in FM0 that has been hydrated for 28 days (Figure 3). The KH_2_PO_4_ and excess MgO powders in PMPC paste undergo chemical reaction under water saturation conditions, and the newly formed MgKPO_4_·6H_2_O fills the pores of the hardened paste [31,32,33]. However, this process ends once the unreacted KDP is consumed. SO_4_^2−^ penetrated into hardened PMPC paste will combine with positive ions in the pore solution and form sulfate crystals (example for MgSO_4_·7H_2_O, Figure 3), which will fill up the pores of the PMPC paste [17,18,19]. The filling effects induce a denser structure in the PMPC paste, preventing the diffusion of shallow SO_4_^2−^ into deeper layers and improving the strengths of PMPC specimens. When a large amount of MgKPO_4_·6H_2_O and sulfate crystals are produced, the filling function will cause volume expansion of the PMPC test pieces.
b. When PMPC test pieces are in a water-saturated state for a long time (for example, for longer than 180 days), the potassium ion on the surface of the MgKPO_4_·6H_2_O crystals will dissociate from K-O bonds and spread into the solution (*Ksp* = 2.4 × 10^−11^) [23,31]. The constitution water in MgKPO_4_·6H_2_O crystals is readily replaced by adjacent free water molecules due to the weak hydrogen bonds, leading to loss of stability in MgKPO_4_·6H_2_O crystals [31,32]. The dissociation of potassium ions and the replacement of bound water lead to the formation of surface fissures in MgKPO_4_·6H_2_O crystals; this process is usually called “dissolution of MgKPO_4_·6H_2_O crystals” [23,31,32,33]. Because of the lack of K^+^, the ions in the pore solution of PMPC paste precipitate out as mineral Mg_3_(PO_4_)_2_·22H_2_O crystals (*Ksp* = 8.0 × 10^−24^) (Figure 3) [23,31]; this process is usually called for “phase transition of MgKPO_4_·6H_2_O crystal”. The “dissociation and phase transition of MgKPO_4_·6H_2_O crystal” can cause deterioration in the pore structure of the PMPC paste (Figure 7 and Table 4) [31,32,33] and accelerate the permeation of SO_4_^2−^ into the PMPC paste [23], causing volume expansion of specimens and reducing the strength of the specimens [18,23].
c. With the dissolution of the MgKPO_4_·6H_2_O crystals in hardened PMPC paste, the unreacted MgO grains exposed continue to hydrolyze into nongelatinous Mg (OH)_2_ crystals (Figure 3; the process is usually called “the slaking of MgO” [23,31]), which weakens the adhesion between unreacted MgO grains and MgKPO_4_·6H_2_O crystals and leads to the structural degradation of the PMPC paste (Figure 7 and Table 4) [16,31,32,33]. The slaking of MgO can accelerate the diffusion of SO_4_^2−^ into PMPC paste, cause rapid volume expansion of specimens [18], and reduce the strength of the specimens [16,23]. Synchronizing with the slaking of MgO grains, the SO_4_^2−^ combines with Mg^2+^ in the hardened paste to generate MgSO_4_·7H_2_O, which will consume some magnesium hydroxide, and the generated MgSO_4_·7H_2_O crystals will fill the capillary pores in the PMPC paste. The effect can ease the deterioration of the structure (Figure 7 and Table 4) but can still cause volume expansion of specimens [16,18].
d. Through the ball-rolling effect of spherical particles in FA, the fluidity of the PMPC paste with some FA was improved, resulting in a decrease in the W/C of the paste with the same fluidity (Table 2) [13]. Some FA can optimize the particle size distribution of dead burnt MgO powders (Figure 1), causing the alkali component particles in the PMPC paste to be more closely piled up [14,15,16,17]. The Al element in FA dissolves and participates in the hydration reaction in an acidic solution, generating AlPO_4_ (Figure 3). The permeation of the Al and Si elements in FA leads to an obvious change in the morphology of MgKPO_4_·6H_2_O in the hardened paste (Figure 5c). All the above-mentioned effects make the structure of FM2 test pieces tend to be dense (Figure 5c), leading to an improvement in the 28-day strength of the specimens (Table 2). Due to it not being easy to penetrate the FM2 test piece with the corrosive medium, its initial resistance to sulfate attack is enhanced compared to the FM0 test piece. The SO_4_^2−^ that penetrated to the hardened paste will combine with Mg^2+^ in the pore solution, forming MgSO_4_·7H_2_O (Figure 3) and filling in the defects in the hardened paste, refining its pore structure (Figure 7). These effects will hinder the diffusion of SO_4_^2−^ in the hardened paste and decrease the strength change and volume expansion of the P2 specimens [18].

## 5. Conclusions

In this work, the sulfate diffusion behavior and law of FA-PMPC paste in a sulfate solution immersion environment was investigated for the first time. By integrating the results of macroscopic measurements, microscopic analysis, and theoretical analysis, its sulfate corrosion mechanism was elucidated. The following conclusions were drawn:

a. In PMPC specimens subjected to one-dimensional SO_4_^2−^ corrosion, the relation between the diffusion depth of SO_4_^2−^ (*h*) and the SO_4_^2−^ concentration (*c* (*h*, *t*)) can be referred by a polynomial very well (the correlation coefficients ≥ 0.999). Under the same conditions, the *c* (*h*, *t*) and *h*_0_ (depth measured when sulfate content is 0) in the FM2 specimen containing fly ash were less than those of the FM0 specimen (reference). At 360-day immersion ages, the *h*_0_ value of the FM0 and FM2 specimens was 14 mm and 12 mm, respectively.
b. The *D* (SO_4_^2−^ diffusion coefficient) of the FM0 and FM2 specimens in different corrosion ages was in all cases on the order of 10^−7^ mm^2^/s (one order of magnitude lower than the Portland cement concrete). Under the same conditions, the surface SO_4_^2−^ concentration *c* (0, *t*), the SO_4_^2−^ computed corrosion depth *h*_00_ and *D* of the FM2 specimen were all lower than those of the FM0 specimen. At 360-day immersion ages, the *c* (0, 360 d) and *h*_00_ in the FM2 specimen (0.2258% and 11 mm) were obviously smaller than that in the FM0 specimen (0.2613% and 14 mm), and the *D* of FM2 specimen (4.38 × 10^−7^) was 64.2% of the FM0 specimen (6.08 × 10^−7^).
c. The strengths of the FM2 specimens soaked for 2 days (the benchmark strength) were larger than those of the FM0 specimens. The strengths of the PMPC test pieces first rose and then fell with the corrosion age, and the strength peaks were attained at the corrosion age of 180 days. The strength change of the FM2 specimens was significantly lower than that of the FM0 specimens. At 360-day immersion ages, the residual flexural/compressive strength ratios (360-day strength/benchmark strength) of the FM0 and FM2 specimens (95.6%/99.2% and 96.7%/98.3%) were all higher than 95%).
d. The volume linear expansion rates of the PMPC specimens continued to increase with the immersion age, and at 360 days the volume linear expansion rates of FM0 and FM2 specimens were 30.61 × 10^−4^ and 15.14 × 10^−4^, respectively. The volume linear expansion rate of the FA-modified FM2 specimen was only 49.5% of that of the reference specimen FM0.
e. This study only investigated the sulfate diffusion law and corrosion mechanism of FA-PMPC paste in a sulfate immersion environment. Research on the sulfate diffusion law of the FA-PMPC system under sulfate freeze–thaw and sulfate dry–wet cycle conditions has not yet been conducted. Subsequent research will continue in this area.

## Figures and Tables

**Table 1:** Chemical components of MgO and FA (wt%).

Chemical Composition		MgO	SiO_2_	CaO	Fe_2_O_3_	Al_2_O_3_	Loi *	Others
Content/%	MgO powder	91.85	3.68	3.14	0.865	0.17	0.12	0.175
	FA	0.935	42.26	5.149	4.026	34.7	8.95	3.98

* Loss on ignition.

**Table 2:** Mix proportions (mass ratio), fluidity, FS, and CS of PMPC paste.

Symbol Name	*m*_FA_/ *m*_(MgO+FA)_	*m*_(MgO+FA)_ /*m*_KDP_	*m*_CR_/ *m*_(MgO+FA)_	W/B	Fluidity (mm)	FS (MPa)		CS (MPa)	
						72 h	28 d	72 h	28 d
FM0	0	3:1	0.13	0.115	160	9.7	12.1	48.6	62.8
FM1	10%			0.110	158	10.2	12.8	54.9	64.9
FM2	20%			0.113	161	11.0	13.9	62.2	69.4
FM3	30%			0.116	158	10.4	13.0	59.1	66.5

FS—flexural strength; CS—compressive strength.

**Table 3:** The status, project, instrument, and analysis conditions for microanalysis of samples.

Sample Status (Quantity)	Project	Instrument	Analysis Conditions
small piece (one piece)	SEM	field emission scanning electron microscope, Nova Nano SEM 450, FEI Corporation, Hillsboro, OR, USA	gold spraying treatment
small piece (one piece)	EDS	X-ray energy spectrum analyzer, AZtec X-MaxN 80, Oxford Company, Abingdon, UK	
powder (3 g)	XRD	X-ray diffraction analyzer, D/max-RB, Rigaku, Tokyo, Japan	scanning range of 5–80°, and a scanning speed of 10°/min
powder (5 mg)	TG DTG	thermal analyzer, STA 409 PC/PG, Netzsch, Selb, Germany	Using nitrogen as a protective gas
small piece (3 piece)	MIP	fully automatic porosity analyzer, PoreMaster-60, Boynton Beach, FL, USA	low pressure 55 psi and high pressure 40,000 psi

**Table 4:** Elemental composition in the microregions of SEM images.

Element	Atomic Percentage/%						
	Area A	Area C	Area D	Area E	Area F	Area G	Area H
O K *	68.95	76.30	58.84	60.68	59.17	77.76	57.79
Na K	2.82		4.35	1.91			
Mg K	9.02	17.55	16.95	10.61	0.99	10.19	9.29
P K	9.65	3.11	8.02	11.11		3.28	4.20
K K	9.55	3.05	8.51	7.51		2.42	3.16
Ca K	-		-	0.19	4.50	6.35	2.02
Al K				2.46	15.49		
Si K				5.36	18.75		1.27
Fe K				0.16	1.09		
S K	-		3.33				
C K							22.27

* K represents the electron shell.

**Table 5:** Pore structural parameters of PMPC samples.

Code and Corrosion Condition	Total Porosity/%	Pore Volume Distribution/%		
		<50 nm	50–200 nm	>200 nm
FM0, 28 d hydration ages	8.96	49.65	9.21	41.14
FM0, 5%Na_2_SO_4_ soaking for 360 d	12.52	32.30	5.43	62.17
FM2, 28 d hydration ages	6.75	35.93	9.97	54.10
FM2, 5%Na_2_SO_4_ soaking for 360 d	8.10	57.54	6.09	36.37

**Table 6:** Calculation results of fitting parameters for SO_4_^2−^ diffusion in FM0 and FM2 test pieces.

Name	*t* /d	*c_s_* (0, *t*)/%	*c*_0_ (*h*_00_*, t*)/%	*h*_00_ /(mm)	** $erf(h_{00}/2\sqrt{Dt})$ **	*D* /mm^2^/s
FM0	0	0	0	0	-	-
	90	0.1115	0.0035	8	0.9686	3.87 × 10^−7^
	180	0.1784	0.0014	10	0.9804	3.01 × 10^−7^
	270	0.2284	0.0047	12	0.9763	5.81 × 10^−7^
	360	0.2613	0.0060	14	0.9970	6.08 × 10^−7^
FM2	0	0	0	0	-	-
	90	0.0820	0.0008	7	0.9902	2.88 × 10^−7^
	180	0.1304	0.0008	8	0.9939	2.73 × 10^−7^
	270	0.1798	0.0070	9	0.9611	4.07 ×10^−7^
	360	0.2258	0.0080	11	0.9646	4.38 ×10^−7^

**Table 7:** Strengths of PMPC specimens with different sulfate corrosion ages.

Code	Parameters	2 d	90 d	180 d	270 d	360 d
FM0	Flexural strength (MPa)	11.4	14.1	14.1	12.8	10.6
	Coefficient of variation (%)	4.39	5.53	6.74	6.40	6.98
	Compressive strength (MPa)	62.5	64.3	69.8	64.0	59.2
	Coefficient of variation	6.08	6.38	6.45	4.69	4.56
FM2	Flexural strength (MPa)	12.3	12.8	13.0	12.4	12.0
	Coefficient of variation (%)	4.88	6.25	6.92	5.65	5.83
	Compressive strength (MPa)	67.2	70.3	72.8	68.3	66.0
	Coefficient of variation (%)	5.80	5.97	6.32	4.69	4.39

## Data Availability

The original contributions presented in this study are included in the article. Further inquiries can be directed to the corresponding authors.
